# The Role of Cytokines in Cholesterol Accumulation in Cells and Atherosclerosis Progression

**DOI:** 10.3390/ijms24076426

**Published:** 2023-03-29

**Authors:** Alexander M. Markin, Yuliya V. Markina, Anastasia I. Bogatyreva, Taisiya V. Tolstik, Deyyara A. Chakal, Denis G. Breshenkov, Eduard R. Charchyan

**Affiliations:** 1 Petrovsky National Research Center of Surgery, 119991 Moscow, Russia; 2 Peoples’ Friendship University of Russia (RUDN University), 117198 Moscow, Russia

**Keywords:** cytokines, cholesterol accumulation, atherosclerosis, inflammation

## Abstract

Atherosclerosis is the most common cardiovascular disease and is the number one cause of death worldwide. Today, atherosclerosis is a multifactorial chronic inflammatory disease with an autoimmune component, accompanied by the accumulation of cholesterol in the vessel wall and the formation of atherosclerotic plaques, endothelial dysfunction, and chronic inflammation. In the process of accumulation of atherogenic lipids, cells of the immune system, such as monocytes, macrophages, dendritic cells, etc., play an important role, producing and/or activating the production of various cytokines—interferons, interleukins, chemokines. In this review, we have tried to summarize the most important cytokines involved in the processes of atherogenesis.

## 1. Introduction

Atherosclerosis is a progressive multifactorial chronic disease characterized by the accumulation of lipids in the wall of large arteries. This process leads to the formation of atherosclerotic plaques, which cause narrowing of the artery lumen and disrupt the patency of the vessel. All this leads to an increased risk of acute conditions, such as myocardial infarction and stroke. In general, cardiovascular diseases (CVD) are the main cause of death in the world [1].

Unfortunately, the aging process is the biggest risk factor for health disorders in general and the malfunctioning of cardiovascular system in particular. The CVD is the cause of death for 40% of people over 65 [2]. However, there is strong evidence that CVD affects young people too, and the severity and scale of the pathological process increases with age [3]. Numerous studies have reported that the subclinical form of atherosclerosis is often present in a large population of young people susceptible to risk factors for atherosclerosis [4]. In such a group there exists a difference in the etiology and profiles of risk factors compared to older patients, which leads to differences in disease progression, prognosis and treatment. The development of atherosclerotic lesions in young people can be explained by the assumption that they smoke more often, they have more other bad habits, they may not take care of their health, some men are obese, and patients from this group may also have a burdened family history [5,6]. There are also unique situations in which, for example, the accelerated development of atherosclerosis and an increased risk of acute myocardial infarction in persons abusing prohibited drugs are shown [7].

Most recent studies confirm the hypothesis that atherosclerosis is a kind of chronic inflammatory disease with an autoimmune component. The pathological process starts with atherogenic modification of low-density lipoproteins (LDL) and their subsequent deposition in the vessel wall, where they can be captured by phagocytes, as well as attacked by specific T cells and antibodies. To summarise the formation of atherosclerotic plaques is initiated by a combination of endothelial dysfunction, chronic inflammation, genetic predisposition and long-term exposure to risk factors, which include hyperlipidemia, hypertension, smoking, male sex and diabetes mellitus [8,9,10].

A well-known and clinically justified risk factor for atherosclerosis is the high content of low-density lipoproteins in plasma. With the accumulation of low-density lipoproteins in the subendothelial space of the arterial wall, their gradual oxidation occurs with the formation of modified low-density lipoproteins [11]. This provokes the onset of an inflammatory reaction. It is characterized by excessive formation of chemotactic and proinflammatory proteins that attract monocytes and other immune cells into the inflamed artery wall. Under the influence of colony-stimulating macrophage factor (M-CSF) and possibly other differentiation factors, most monocytes in early atheromas become macrophages and/or dendritic cells. After monocytes have differentiated into macrophages, they excessively absorb modified LDL and may transform into foam cells. This leads to the formation of atherosclerotic plaques [12]. Further, macrophages continue to populate the atherosclerotic plaque by attracting new cells from the vascular bed and proliferation of resident cells located in the surrounding tissues. It is noteworthy that macrophage proliferation signals, such as IL-4 [13], have been found in atherosclerotic plaques, especially in macrophage-rich fatty streaks, where they co-localize with foam cells [14].

In addition to macrophages, an important role in the development of the pathological process is played by dendritic cells (DCs), which also have a monocytic origin and belong to the cells of innate immunity [15]. DCs are professional antigen-presenting cells that initiate the immune response. DCs are present in a healthy vessel, but there is a directly proportional relationship between their number and the stage of development of the disease [16]. These data show the fundamental role of inflammation in atherogenesis and its connection with the accumulation of lipids. This relationship is expressed in the ongoing process of capture by macrophages of modified LDLs from the extracellular space of intima. Inside macrophages, cholesterol undergoes hydrolysis in lysosomes. Next, cholesterol molecules exit the lysosomes and are esterified in the cytoplasm, where they accumulate in the form of cytoplasmic lipid droplets. This process completes the formation of a foam cell. Active formation of foam cells occurs in the early stages of the development of atherosclerotic lesions. In the later stages of lesions, foam cells are destroyed and release their contents into the extracellular space, repeatedly provoking the development of inflammation and causing the development of structural instability of the plaque [17].

The processes of foam cell formation can be triggered by unregulated absorption of modified LDL through receptors (for example, CD36) and lead to an uncontrolled increase in cholesterol. This destabilizes lysosomes, disrupts the metabolism of cholesterol and fatty acids, and activates the NLRP3 (NLR family pyrin domain containing 3) inflammasome, and then the production of IL-1ß. Further, macrophages, penetrating into the emerging atherosclerotic plaques, support local inflammation by producing reactive oxygen species (ROS) and secreting inflammatory cytokines and chemokines, including TNF-α, IL-1β, IL-6, IL-8 and TGF-β, which attract additional immune cells [18,19].

The inflammasome is cytoplasmic multi-protein complex containing sensory protein, inflammatory caspases and, in some cases, an adapter protein connecting them. They can be activated by a set of endogenous and exogenous stimuli, which leads to enzymatic activation of canonical caspase-1 [20], non-canonical caspase-11 [21] or equivalent caspase-4 and caspase-5 in humans [22] or caspase-8 [23]. This leads to the secretion of pro-inflammatory cytokines IL-1β and IL-18, and in some cases, apoptotic and pyroptotic cell death is triggered. The activation of inflammasome is a vital process. It is necessary to fight microbial pathogens or the consequences of tissue damage. At the same time, impaired activation of inflammasomes can cause uncontrolled tissue reactions, which may cause various diseases, including auto-inflammatory disorders, cardiometabolic diseases, cancer and neurodegenerative diseases. Therefore, it is important for the organism to maintain a delicate balance between the activation and inhibition of inflammasome assembly. Recently, there has been an increasing number of studies of the structural and molecular mechanisms underlying the regulation of the transmission of signals by inflammasomes [24].

Next, we will try to characterize the cytokine stimuli involved in the development of the described processes as fully as possible. The features of the proatherogenic or antiatherogenic action of pro-inflammatory and anti-inflammatory cytokines and regulatory molecules will be considered.

## 2. What Are Cytokines?

Here we describe the most important groups of cytokines in terms of their participation in the processes of atherogenesis. Cytokines are a set of small proteins involved in cellular signaling pathways. The cytokine superfamily is produced by various cells of the body, and includes interferons, interleukins (IL-1, IL-6, colony-stimulating factors (CSF), transforming growth factors (TGF), tumor necrosis factor (TNF) family, chemokines and others. Cytokines can affect all stages of atherosclerosis development [25]. They are produced by T cells [26], monocytes [27], macrophages [28], platelets [29], endothelial cells (EC) [30], smooth muscle cells (SMC) [31], pericytes [32], adipocytes [33] and others in response to inflammation and other stimuli. Increased production of proinflammatory cytokines is associated with the progression of the disease and contributes to the development of atherosclerosis. In the later stages of atherosclerosis, proinflammatory cytokines contribute to the destabilization of atherosclerotic plaques by provoking apoptosis of plaque cells and matrix degradation. This leads to the atherosclerotic plaque ruptures and the formation of blood clots [34].

### 2.1. Interferons

Interferons (IFN) are the most important cytokines with antimicrobial, antitumor and immunomodulatory activity. The three types of IFN (I, II, and III) are classified by their receptor specificity and sequence homology. IFNs are produced and secreted by cells in response to certain stimuli. IFNs were discovered in 1957 by Isaacs and Lindenmann in a study called “Virus interference”. They identified a new factor that can prevent the virus from entering cells, and called it “interferon” [35]. To date, three types of IFN have been identified based on the classification of their specific receptors. In humans, the IFN type I (IFN-I) family consists of 13 IFNα subtypes and one each of IFNβ, ε, κ, ω subtypes. Type II IFN (IFN-II) includes only IFNγ, and IFN type III (IFN-III) includes IFN-λ1 (IL-29), IFN-λ2 (IL28A), IFN-λ3 (IL-28B) and IFN-λ4 [36].

All interferons (IFNs) transmit signals via the JAK/STAT path. Interaction with receptors induces sequential activation of JAK (janus kinases), TYK (tyrosine kinase) and STAT (signal transducer and activator of transcription), which leads to the activation of ISG (IFN-stimulated genes) transcription. In the absence of stimuli, the cytoplasmic domain of the IFN receptor is bound by inactive JAK kinases. After IFN binding, these JAKs undergo phosphorylation and are activated, which leads to phosphorylation of the IFN receptor and a change in the position of STAT proteins. These STAT, now located next to the activated JAK, are phosphorylated by tyrosine and released from the receptor. Activated STATS undergo homo- or heterodimerization and nuclear translocation. STAT1-2 heterodimers bind to IRF9 (IFN-regulatory factor) to form the active transcription complex ISGF3, whereas STAT homodimers are direct transcription activators. As a rule, type I and type III IFN signaling activates TYK2 and JAK1, which leads to STAT1-2 heterodimerization and ISGF3 formation, whereas type II IFN signaling activates JAK1 and JAK2, causing STAT1 homodimerization [37,38,39,40,41].

#### 2.1.1. The Type-I Interferons (IFNs)

The type I IFNs includes IFN-α, β, ε, κ and ω. The genetic loci of these subtypes contain different regulatory elements that probably contribute to differentiated control during signal transmission. These subtypes also exhibit different affinity of binding to the receptor, which contributes to various strength of signal transmission. Type I IFNs transmit a signal through a ubiquitously expressed heterodimeric receptor consisting of IFNAR1 and IFNAR2 subunits. The type I IFNs activates the transcription factor ISGF3, which binds to IFN-stimulated response elements (ISREs) inside ISG promoters. It should be added that the priming effect is noted for IFN, due to which small amounts of IFN maintain high basal STAT1/2 and IRF9 levels. This allows immune cells to respond quickly to the IFN signal. In certain cell types, other members of the STAT family (STAT3, 4, 5A/5B and 6) can also be activated by the type I IFNs. In general, the type I IFNs are the major component of antimicrobial immune defense, and ISG-induced products act by limiting infection and modulating/enhancing the adaptive immune response. Most cell types can respond to the type I IFNs signaling [42,43].

#### 2.1.2. The Type-II Interferons

IFN type II refers to IFN-γ, which binds as a dimer to the IFNGR (interferon gamma receptor) complex, consisting of two IFNGR1 subunits and two IFNGR2 subunits. Activation of the receptor induces phosphorylation of JAK1 and JAK2, which makes it possible to combine STAT1 subunits. The STAT1 pair is phosphorylated, homodimerized and moved to the nucleus, where it binds gamma activated sequence (GAS) elements inside ISG promoters. GAS elements can also be activated by the STAT complex, which was induced by type I interferon. Despite the fact that IFN-γ production is limited to hematopoietic cells, IFNGRS are widely represented in all cell types, so they are able to respond to IFN-γ signals. IFN-γ signals can induce the expression of genes that initiate a type I IFN response. Similarly, ISG products from type I IFN signaling can enhance IFN-γ signaling. In general, IFN-γ plays a key role in the regulation of immune function, including the polarization of the T-cell response and activation of myeloid cells, as well as the binding of innate and adaptive immune responses [44,45].

#### 2.1.3. The Type-III Interferons

**Figure 1:**
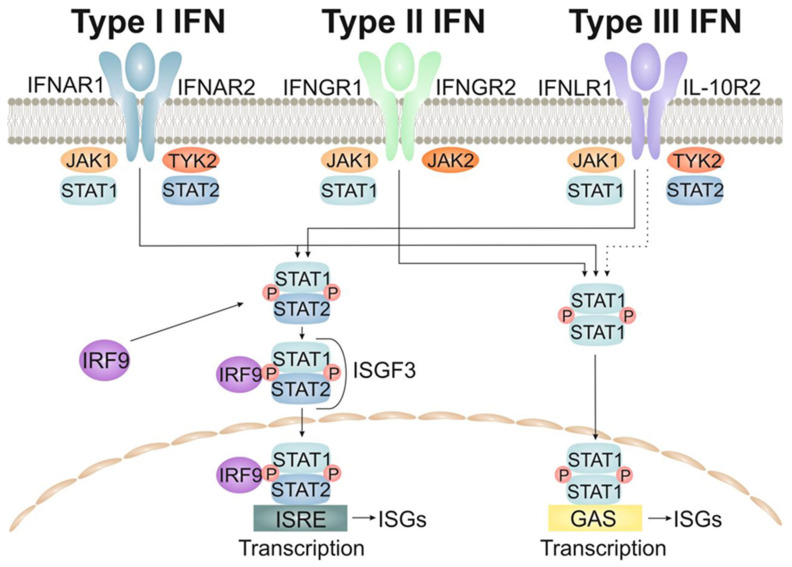
Interferon signaling pathways to the cell nucleus.

Type III IFNs include IFNλ1, IFNλ2, IFNλ3 (first called IL-29, IL-28A and IL-28B) and the recently described IFNλ4. IFN-λ binds to a heterodimeric receptor consisting of IFNLR1 (also known as IL-28Ra) and IL-10R2. While IL-10R2 is widespread, the expression of IFNLR1, which is uniquely used by type III interferons, is limited to epithelial cells, subpopulations of myeloid cells and some nerve cells. Thus, it is believed that type III interferons act mainly on barrier surfaces, including mucous membranes and the blood-brain barrier [46]. The complex of IFN-λ and its receptor induces the same ISG as type I IFN via ISGF3 (interferon-stimulated gene factor 3). This serves as an indirect confirmation that IFN-λ signaling uses similar components of the JAK/STAT path that use the type-I interferons. In addition, data has recently been obtained that JAK2 can mediate the transmission of type III IFN signals [47]. The transmission of type I and type III IFN signals demonstrates different kinetics, and it has been shown that these interferons work in dynamic balance, complementing each other’s work [48,49,50]. The main pathways for signaling to the cell nucleus by the interferons are shown in Figure 1.

### 2.2. The Interleukins

**Figure 2:**
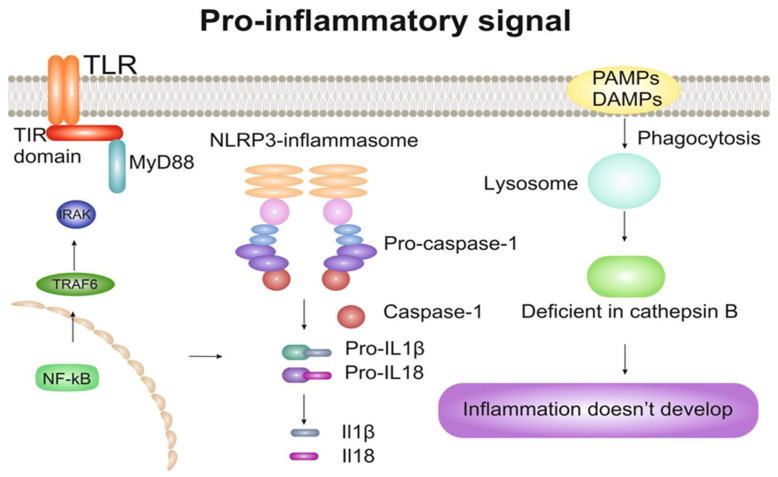
Pro-inflammatory signaling pathways in the cell.

The first and most important group of interleukins to be mentioned is the Il-1 family. The interleukin-1 (IL-1) cytokine family comprises 11 members: IL-1α, IL-1β, IL-1 receptor antagonist (IL-1Ra), IL-18, IL-33 and IL-1F5–IL-1F10. The biology of IL-1F5–IL-1F10 is less well characterized than that of IL-1, IL-18 and IL-33 [51]. The signal initiation mechanism is a step-by-step process in which a cytokine binds a related receptor. Next, the cytokine-receptor complex activates a secondary receptor. Intracellular domains of Toll/IL-1 receptors (TIR) are in close proximity, initiating a cascade of NF-kB signal transmission (Figure 2). Due to the strong inflammatory response caused by IL-1 family cytokines, there are several physiological mechanisms for inhibiting IL-1 family signaling, including cytokine antagonists and trap receptors [52]. As described above, two members of this family, namely IL-1β and IL-18, are the first to be released from the activated immune cell. The secretion of these cytokines is mediated by the caspase-1-activating NLRP3 inflammasome and the subsequent development of inflammation. It was found that cholesterol crystals are able to activate the NLRP3 inflammasome in phagocytes in vitro. In addition, studies on mice deficient in NLRP3 inflammasome components, cathepsin B, cathepsin L, or IL-1 molecules showed that intraperitoneal injection of cholesterol crystals did not develop acute inflammation, unlike mice without this deficiency [19]. Activation of caspase 1 leads to the activation of NF-kB (nuclear factor kappa-light-chain-enhancer of activated B cells) and the induction of pro-IL-1β transcription, the expression of pro-IL-18 comes constitutively and increases under conditions of pro-inflammatory activation. The secretion of cytokines IL-1β and IL-18 promotes the activation of other immune cells to the site of inflammation [53].

Here we will confine ourselves to describing the signaling of the interleukin-1 (IL-1) cytokine family as a fundamental example of the work of all cytokine families. We will not add a description of other groups of cytokines, but in the chapters devoted to the specific properties of certain groups we will describe each cytokine of interest to us.

### 2.3. The Tumor Necrosis Factor Superfamily

The next important group is the tumor necrosis factor superfamily (TNFSF). Numerous members of the TNFSF have recently shown emerging roles in both the protection and progression of coronary and peripheral artery disease. The cause of these diseases is atherosclerosis. The most important role in the development of atherosclerosis is TNF-α, TNF-related apoptosis-inducing ligand (TRAIL), TNF-like weak-inducer of apoptosis (TWEAK), CD40L, and their cognate receptors.

**Figure 3:**
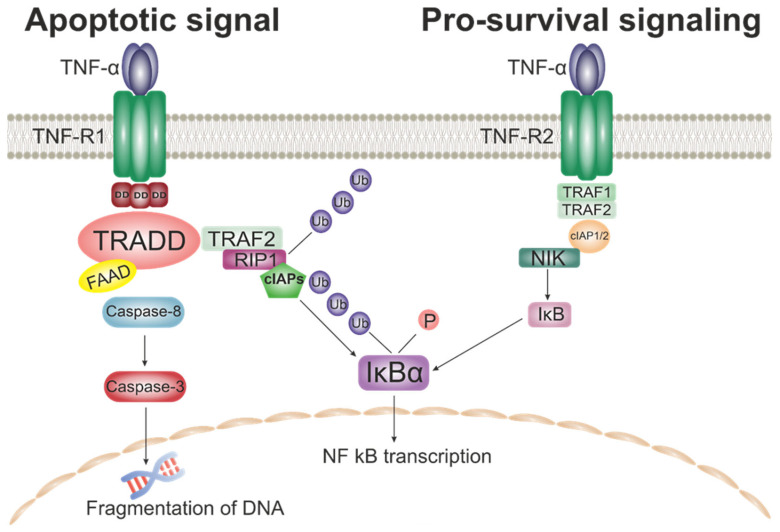
Possible options for transmitting a signal of TNF to the cell.

Signals transmitted by TNFSF members inside the cell are capable of inducing both pro-apoptotic signals and survival-promoting signals [54] (Figure 3). These signal paths are common to TNF-α, TRAIL, TWEAK, and CD40. TNFSF signaling occurs through the TNF-R1 and TNF-R2 receptors. Moreover, TNF-R1 refers to the DD-containing subgroup of TNFR, which mediate apoptosis [55].

Here it is worth mentioning in general about the proteins of the superfamily of death domains (DD) and in general about the work of DD mediated TNFSF signaling. This pathway plays a central role in signaling apoptosis and inflammation through the formation of oligomeric complexes. This superfamily is composed of the DD, Caspase Recruitment Domain (CARD), Death Effector Domain (DED), and Pyrin Domain (PYD) subfamilies. These protein subfamilies are capable of forming homotypic interactions, which facilitates the subsequent assembly of oligomeric signaling complexes. Their main function is to promote the activation of pro-apoptotic caspases. This process can occur through the release of Cytochrome C from mitochondria, which triggers the oligomerization of Apaf-1 (apoptotic protease activating factor 1). Apaf-1 is then able to bind to Caspase-9 through the interaction of two CARDs and promote its activation through dimerization and subsequent autocatalytic cleavage [56]. Also, activation of pro-apoptotic caspases can begin along the TNFR associated death domain (TRADD) recruitment pathway. To initiate apoptosis, TRADD binds to a second adaptor protein called Fas-associated protein with death domain (FADD) causing caspase-8/10 activation, triggering a series of caspase cleavages that culminate in cellular apoptosis. The FADD complex in combination with procaspase-8/10 is called the death-inducing signaling complex (DISC) [57,58,59,60].

On the other hand, the binding of TNF-α to the receptor can lead to trimerization and binding of TRADD to DD. The resulting complex then helps attract additional proteins: TNF receptor associated factor 2 (TRAF2) and receptor-interacting protein-1 (RIP1). The activation of the cellular inhibitor of apoptosis proteins (cIAPs) with TRAF2 (TNF receptor associated factor 2) leads to the ubiquitination of RIP1, which allows the complex to be associated with the transforming growth factor β-activated kinase-1 (TAK1). The resulting complex stimulates the translocation and activation of NF-κB, while the stimulation of NF-κB follows either the canonical or non-canonical pathway. Canonical activation of NF-κB causes degradation of the NF-κB inhibitor (IκB) through the IκB kinase complex (IKK) [61], whereas the non-canonical pathway depends on the stimulation of IKKα by the kinase NF-κB (NIK) [62]. Canonical stimulation appears to be more common, and this is the pathway by which the TNF-R1 signal is transmitted. The complexity and diversity of signaling pathways do not allow us to consider in this review all possible options for transmitting a signal to the cell. However, even this small excursion into the theory of TNFSF signaling already suggests the existence of a direct relationship between the change in the level of cytokines of this group and the development of atherosclerotic lesions. This is confirmed by both clinical and laboratory studies, including on model animals [63,64,65,66].

### 2.4. Chemokines

These are a large family of structurally related, chemoattracting cytokines that are divided into subgroups based on the position of the amino-terminal cysteine residues (CC, CXC, CX3C, XC). Chemokines are divided into four groups: two large families represented by a plurality of members, these are the chemokines CXC and CC, as well as two groups with a single representative in each group: fractalkine (CX3C) and lymphotactin (C). Chemokines CC contain four cysteine and are of particular interest as chemotactic monocyte proteins and eotaxin belong to this group. All chemokines play crucial roles in many forms of CVD. Chemokines interact with receptors that activate heterotrimeric G proteins and associated intracellular signaling pathways [67,68,69]. Chemokines and their receptors are widely expressed in vascular cells, such as ECs, SMCs, leukocytes [68].

The main role of chemokines is to control the migration of leukocytes. This is of great importance in the pathogenesis of atherosclerosis, where one of the leading roles is played by the interaction between leukocytes and endothelial cells of blood vessels. These ratios are mediated through selectins, which are constitutively expressed on white blood cells (L-selectins, CD62L) or endothelial cells (E- and P-selectins, CD62E and CD62P). The expression of selectins is induced by inflammatory mediators. The process of penetration of leukocytes through the endothelial barrier is as follows: integrin molecules on leukocytes (e.g., CD11/CD18) are activated by cytokines and chemotactic factors and bind to adhesion molecules on the cell surface of endothelial cells. And then leukocytes migrate through the endothelium into the tissues in response to a gradient of chemotactic factors. Chemokines are induced in response to exogenous (e.g., microorganisms, toxic components) or endogenous (e.g., pro-inflammatory cytokines) signals. Chemokines also stimulate tissue-infiltrating leukocytes to produce proteolytic enzymes, which facilitates the migration of cells through the basement membrane and extracellular matrix [70].

The first group of chemokines we wanted to talk about are CC-chemokines (or β-chemokines). They are a family consisting of 28 chemotactic cytokines with an N-terminal CC domain. These are, for example, human chemokines: CCL1, CCL3, CCL4, CCL5, CCL18, CCL19, CCL20, CCL21, CCL25, CCL27 and CCL28; Chemokine receptors with CC motif: CCR5, CCR6, CCR7, CCR8, CCR9 and CCR10. Molecules of this group regulate the work of CD4+ and CD8+ lymphocytes, dendritic cells, eosinophils, macrophages, monocytes and NK cells. They are actively involved in proliferation, apoptosis resistance, drug resistance, migration and invasion of cancer cells [71]. However, there is greater heterogeneity within CC chemokines, not only with respect to the individual spectrum of action, but also in their protein sequences and chromosomal localization. As mentioned above, chemotactic monocyte proteins (MCPs) and eotaxin, based on structural and functional characteristics, belong to the subfamily CC-chemokines. Four human MCPs (MCP-1–4) have currently been described. The most well-characterized is the chemokine monocytic chemoattractant protein-1 (MCP-1/CCL2). In particular, MCP-1 is a powerful chemokine that attracts blood monocytes to the site of inflammation, tumor or atherosclerotic lesions [72].

The next important group of chemokines are members of the CXC family. This group is important for us precisely in the context of the development of cardiovascular diseases, as many of them contribute to angiogenesis. These are the following representatives: CXCL1, CXCL2, CXCL3, CXCL5, CXCL6, CXCL7, CXCL8. Angiogenic factors in the local microenvironment can act in different ways. For example, the first step may involve the activation of endothelial cells by vascular endothelial growth factor (VEGF), which leads to the activation of the anti-apoptotic molecule Bcl-2, which promotes the expression of CXCL8 by endothelial cells. Increased expression of CXCL8, in turn, affects the formation of the angiogenic phenotype of endothelial cells by the autocrine and paracrine routes. CXCL8 helps improve endothelial cell survival and proliferation [73,74,75]. There is evidence confirming the participation of CXCL cytokines in the remodeling of myocardial damage after a heart attack [76].

Fractalkine (CX3CL1) is a large cytokine protein of 373 amino acids, it contains several domains and is the only known member of the CX3C family of chemokines. The structure of the polypeptide differs from the typical structure of other chemokines. For example, the distance between the characteristic N-terminal cysteines is different, there are three amino acids separating the initial pair of cysteines in CX3CL1. Fractalkin was first discovered in 1987 Bazan et al. [77]. It is not only the only member of the CX3C subfamily, but also an unusual chemokin with a dual function. Fractalkin acts either in soluble form or as an adhesion molecule. Soluble CX3CL1 has a potent chemoattractant effect on T cells and monocytes, while cell-bound chemokine promotes strong leukocyte adhesion to activated endothelial cells, where it is predominantly expressed. CX3CL1 exhibits its adhesive and migratory functions by interacting with the chemokine receptor CX3CR1 [78].

XCL1, or lymphotactin (Ltn), is a member of the C-class chemokine, predominantly expressed by T cells, NT cells, synovial macrophages, and dendritic cells. This chemokine exhibits chemotactic and immunomodulatory activity against T cells, natural killer (NK) and macrophages, and plays an important role in the cytotoxic immune response mediated by dendritic cells. At present, its role in the development of arthritis and progressive bone degradation in rheumatoid arthritis is well studied [79].

## 3. How Cytokines Impact Cholesterol Metabolism

Next, we will describe the features of the influence of cytokines of interest to us on the role mediated through cholesterol metabolism and inflammation in the initiation and progression of atherosclerosis. And here and further we will adhere to the same sequence in the description of proteins as in the previous chapter.

### 3.1. Impact of Interferons on Cholesterol Metabolism

In the original work [80] interferon type I (IFN) signaling has been shown to alter the balance of lipid metabolism programs by reducing synthesis and increasing the uptake of cholesterol and long-chain fatty acids in the cell. As it turned out, the physiological meaning of this effect is explained by the fact that the modulation of these metabolic pathways in macrophages is necessary for the development of resistance to viral infection. It has been shown that spontaneous restriction of lipid uptake by biosynthesis stimulates the IFN response of type I. And the activation of the response along the IFN pathway of type I is associated with a decrease in the size of the pool of synthesized cholesterol and is inhibited by replenishing cells with free cholesterol. Thus, IFN signaling reduces cholesterol biosynthesis, and conversely, a sharp decrease in cholesterol biosynthesis causes type I IFN responses and stimulates cells to enhance antiviral immunity.

Interferons are able to mediate abrupt changes in cholesterol metabolism pathways by rapidly reducing cholesterol synthesis, increasing the accumulation of cholesterol esters in lipid droplets, and stimulating the production of cholesterol derivatives such as oxysterol 25-hydroxycholesterol (25HC) [81]. Altering lipid metabolism pathways modulates host defense by blocking viral penetration, regulating innate immunity receptor signaling, immune cell skewing, and increasing the phagocytic capacity of macrophages. This process is seen as a fundamental component of the host’s immune response. At the same time, in the case of violations of the pathway of activation and secretion of IFNs, its excess production can stimulate an immune response without the presence of a pathogen, which can lead to the start of a vicious circle of chronicity of inflammation [82]. It is also worth noting that production of 25-hydroxycholesterol (25-HC) promotes macrophage foam cell formation [83].

Speaking about the effect of IFNs on cholesterol metabolism, it is worth mentioning that IFNs induce several genes involved in the esterification and outflow of cholesterol. Co-incubation of macrophages with interferon increases the formation of cholesterol esters, and it has been found that cholesterol in the culture medium is not required for the accumulation of esterified cholesterol in response to IFN. This indicates that the source of cholesterol for esterification may be derived from the cell membrane rather than from the extracellular environment [81].

### 3.2. Impact of Interleukins on Cholesterol Metabolism

#### 3.2.1. The Interleukin-1 (IL-1) Cytokine Family

Among the numerous inflammatory mediators, cytokines of the IL-1 family play an important role. This group includes several pro-inflammatory cytokines (IL-1α, IL-1β, IL-18, IL-33, IL-36α, IL-36β and IL-36γ) and one anti-inflammatory cytokine (IL-37). According to current evidence, the most studied members of the IL-1 cytokine family are IL-1α, IL-1β, IL-18, and IL-1Ra [84].

Many members of the IL-1 family promote atherogenesis and are important mediators of vascular and systemic inflammation. The key mediator in the production of cytokines of the IL-1 family is the inflammasome—NLRP3. This is one of the main signaling complexes of the innate immune response. NLRP3 consists of: NOD (nucleotide oligomerization domain)-, LRR (leucine-rich repeat)- and PYD-containing protein 3. The assembly of NLRP3 in cells of atherosclerotic lesions is activated by modified low-density lipoproteins and cholesterol crystals. Inflammasome activation occurs in response to pathogen-associated molecular patterns (PAMP), conserved infectious agent compounds, and damage-related molecular patterns (DAMP). PAMP and DAMP are perceived by pattern recognition receptors (PRRs) and innate and adaptive immunity cells [85]. The formation of inflammasomes is induced by several intracellular PRRs. When activated, they form large multimolecular signaling platforms that catalyze the maturation of pro-IL-1β and pro-IL-18 [86]. Thus, this process contributes to the formation of an inflammatory response in the vessel wall, which, in the absence of a stimulus to completion, leads to the development and progression of atherosclerosis. At the same time, in case of impaired functioning of the NLRP3 inflammasome, anti-atherogenic effects can be recorded. So, it was demonstrated that mice with double knockout Apoe^−/−^/Caspase-1^−/−^ showed a decrease in the spontaneous development of atherosclerotic lesions after being fed a chow diet for 26 weeks [87]. A similar study confirms that the accumulation of cholesterol in myeloid cells activates the NLRP inflammasome, and a deficiency of NLRP3 or caspase-1/11 reduces the size of the atherosclerotic lesion in LDLR^−/−^ mice [88].

#### 3.2.2. Interleukin 4

Interleukin-4 (IL-4) and its transcripts have been found in atherosclerotic lesions in both humans and mice. To determine whether this local release of IL-4 affects the metabolism of macrophage lipids, the effect of this cytokine on intracellular cholesterol esterification during incubation with modified LDL was investigated. IL-4 significantly enhanced cholesterol esterification induced by acetylated LDL (AcLDL) in both mouse peritoneal macrophages and the J774 mouse macrophage cell line. This was not a generalized effect on lipoprotein metabolism because IL-4 had no effect on cholesterol esterification in the presence of LDL or beta-VLDL (very low-density lipoproteins). The determination of binding isotherms showed that IL-4 increased the number of AcLDL binding sites on the cell surface. IL-4-induced AcLDL cholesterol esterification was weakened by a Class A scavenger receptor (SR-A) antagonist, fucoidan, and a monoclonal antibody against SR-A, 2F8 in mice [89]. IL-4 is responsible for the expression of peroxide enzymes, for example, human 15-lipoxygenase (ALOX15), involved in the oxidation processes of LDL. This prompted the formation of a hypothesis about the possible direct participation of IL-4 in the development of atherosclerosis. However, studies to date have shown that IL-4 deficiency does not affect early atherosclerosis, at least in C57BL/6 mice fed a high-cholesterol diet [90].

#### 3.2.3. Interleukin 5

Interleukin 5 (IL-5) is a cytokine produced by eosinophils, mast cells, macrophages, CD4+ T, and type 2 innate lymphoid cells (ILC2). Its expression is regulated by several transcription factors, including GATA binding protein 3 (GATA3). IL-5 may play a role in the development of human atherosclerosis. Its level is associated with the plasma concentration of anti-OxLDL (oxidized low-density lipoprotein) antibodies, which may be associated with a decrease in the rate of development of atherosclerosis [91,92]. This is indirectly confirmed by the fact that autoantibodies against IL-5, suppressing its function, accelerate the development of atherosclerosis [93]. And IL-5 overexpression suppresses the development of CVD by reducing inflammation in macrophages [94] and SMC apoptosis [95]. It has also been shown that LXR (liver X receptor) activation induces macrophage IL-5 expression [96]. This may indicate a role for LXR not only in stimulating ABCA1 expression and cholesterol outflow, but also in inducing IL-5 expression in macrophages. IL-5 significantly up-regulated ATP-binding cassette transporter A1 (ABCA1) expression in a dose-dependent and time-dependent manner. As a result, IL-5 increased ABCA1-mediated cholesterol efflux. The regulation of this process occurs through the miR-211/JAK2/STAT3 signaling pathway in THP-1-derived macrophages [97].

#### 3.2.4. Interleukin 6

IL (interleukin)-6 is a pivotal cytokine of innate immunity, which regulates a broad set of immunological functions traditionally associated with host defense, immune cell regulation, proliferation, and differentiation. Enhanced release of this cytokine occurs in response to acute infections, chronic inflammation, metabolic disorders, physiological stress, etc. This, in turn, leads to an increase in the synthesis of acute phase proteins by the liver, activation of endothelial cells, increased coagulation processes, activation of the hypothalamic-pituitary-adrenal system, stimulation of proliferation and differentiation of lymphocytes [98]. In turn, the level of C-reactive protein (CRP) in the blood plasma can be used as a predictor of the risk of developing cardiovascular events along with the assessment of the level of total cholesterol or low-density lipoprotein cholesterol [99,100]. It has also been shown that an increase in the level of IL-6 is associated with impaired mitochondrial function in vascular cells, which may be a factor accelerating the development of atherosclerosis [101]. Given current efforts to prevent and treat atherosclerosis, at a fundamental level, understanding of the relationship between inflammation, cholesterol levels, and cardiovascular risk has not changed significantly since the data described decades ago. This may indicate that future treatments for atherosclerosis will require a combination of inhibiting inflammation and lowering cholesterol [102].

#### 3.2.5. Interleukin 7

IL-7, a member of the type I cytokine subfamily, is involved in the development and proliferation of T- and B-lymphocytes, which play an important role in the functioning of the innate immune system and the inflammatory response [103]. It is known that atherosclerosis is a multifactorial disease involving various pathological mechanisms, including cholesterol accumulation, lipoprotein modification, endothelial dysfunction, and chronic inflammation [104]. IL-7 induces the activation of monocytes and natural killer cells, which induces the production of inflammatory cytokines (IL-1b, IL-8, MCP-1, MIP, etc.), and the expression of chemokine receptors (CCR1, CCR2, CCR5), which are highly expressed in atherosclerotic plaques [105]. IL-7 acts by binding to the heterodimeric IL-7R receptor, which consists of two glycosylated subunits: IL-7Rα (CD127, 65 kDa) specific for IL-7 and a common γ-chain (γc, CD132, 56 kDa) common to IL-2, 4, 7, 9, 15 and 21 receptors. The cytoplasmic domains of IL-7Rα (long, 195 residues) and γc (short, 86 residues) are responsible for binding a large number of proteins involved in signaling pathways that support survival and proliferation cells include Janus kinases, JAK1 and JAK3 (associated with IL-7Rα and γc, respectively), which are involved in the JAK/STAT pathway. In addition, binding of IL-7 to its receptor triggers the MAPK (mitogen-activated protein kinases) and PI3K/Akt pathways, which induce mitogenic and anti-apoptotic signals [106]. PI3K/Akt-dependent and independent activation of NF-kB leads to the recruitment of monocytes/macrophages and plays an important role in atherogenesis [107]. To date, there is evidence that IL-7 may be a potential therapeutic target for the treatment of chronic inflammatory diseases. For example, in animal experiments, IL-7 receptor blockade has been shown to be effective in improving chronic inflammatory diseases by downregulating memory T-cell responses [108]. In addition, IL-7 was found to be one of the three major gene transcripts affected by cholesterol lowering [107].

#### 3.2.6. Interleukin 8

IL-8 is able to induce the development of extracellular immune traps of neutrophils, which, by activating the transmission of NF-κB signals in macrophages, can aggravate the course of atherosclerosis at the cellular level. One study showed that PMA-induced NETosis directly activated the TLR9 (toll-like receptor)/NF-κB pathway in macrophages and stimulated the release of IL-8. This pathway works as follows: IL-8 interacted with its receptor CXC chemokine receptor 2 (CXCR2) on neutrophils, which led to the formation of neutrophil extracellular traps (NETs) through Src and extracellular signal-regulated kinase (ERK) and p38 mitogen-activated protein kinases (MAPK) [109]. Some studies suggest that IL-8 has an important role in the regulation of cholesterol efflux. Later was described IL-8 enhances the expression of miR-183, which then inhibits ABCA1 expression and cholesterol efflux [110]. Cholesterol outflow plays an important role in anti-atherogenesis, and a modification of this process could provide a new therapeutic approach to cardiovascular disease [111].

#### 3.2.7. Interleukin 10

Interleukin (IL)-10 is an anti-inflammatory cytokine produced mainly by macrophages and T lymphocytes of the Th2 subtype. As for atherosclerosis, its main roles include inhibiting the activation of macrophages, as well as inhibiting matrix metalloproteinase, pro-inflammatory cytokines, and cyclooxygenase-2 expression in lipid-laden and activated foam cells of macrophages [112]. The members of the interleukin (IL)-10 family, including IL-10, IL-19, IL-20, IL-22, IL-24, IL-26, and the distantly related IL-28A, IL-28B, and IL-29, play a crucial role in inhibiting inflammation. Increased IL-10 expression by macrophages inhibits atherosclerosis in LDLR (^−/−^) mice by reducing the accumulation of cholesterol esters in cells. Experiments with primary macrophages showed that IL-10 stimulated stimulated both the uptake (by up-regulating scavenger receptors) and efflux of cholesterol (by activating the PPARg (Peroxisome Proliferator-Activated Receptors) -LXR-ABCA1/ABCG1 pathway) [113]. Other studies confirm the findings that the anti-atherogenic properties of IL-10 may include enhancing effects on cholesterol efflux mechanism that involves cross-talk with LXRα activation [114]. In turn, the deficiency of the IL10 receptor (IL-10R1) on myeloid cells leads to polarization of macrophages along the pro-inflammatory pathway in vitro. On the other hand, in in vivo experiments (in a mouse model), there was a significant decrease in the size and severity of atherosclerotic lesions, which was the result of less accumulation of myeloid cells in the lesions. In addition, with a deficiency of myeloid IL-10R1, a significant decrease in plasma and liver cholesterol levels was observed, which was reflected in the lipid content in plaques. This was due to decreased levels of VLDL and LDL, probably in response to decreased cholesterol absorption in the gut. In addition, mice deficient in IL-10R1 showed significantly higher loss of sterol with feces caused by increased outflow of non-biliary cholesterol. The induction of this process was associated with a violation of ACAT2 (acetyl-Coenzyme A acetyltransferase 2 gene) -mediated esterification of cholesterol in the liver and plasma. Thus, clarifying experiments on the anti-atherogenic effect of IL10 are required [115].

#### 3.2.8. Interleukin 12

IL-12 is produced by various cell types such as monocytes, neutrophils, dendritic cells, and macrophages on the activation of these cells by pathogens, by CD40 ligand–expressing T cells, or by extracellular matrix components, such as the glycosaminoglycan hyaluronan. IL-12 is a heterodimeric (p70) cytokine, which consists of a 35-kDa light chain (p35) and a 40-kDa heavy chain (p40) [116]. IL-12 appears to be involved in pro-atherosclerotic reactions by stimulating the migration and adhesion of leukocytes in fat streaks, which was shown in a mouse model of hypercholesterolemia [117].

#### 3.2.9. Interleukin 13

Interleukin 13 (IL-13) is a protein that in humans is encoded by the IL13 gene. IL-13 is a cytokine secreted by T helper type 2 (Th2), CD4 cells, natural killer T cells, mast cells, basophils, eosinophils, and lymphocytes. Described results demonstrating that IL-13 induces changes in cholesterol metabolism in the liver in a rat model leading to hypercholesterolemia [118].

#### 3.2.10. Interleukin 15

Interleukin 15 (IL-15) is a cytokine that belongs to the interleukin-2 (IL-2) family and may play an important role in the development of an innate and adaptive immune response. The structure of IL-15 is partially similar to IL-2, they have some common biological effects including immunoregulation. IL-15 levels are elevated in some cardiovascular diseases, such as myocardial infarction and atherosclerosis. At the same time, there is evidence demonstrating that IL-15 has a protective effect in myocardial infarction and myocarditis, reducing the death of cardiomyocytes [119]. So, in viral-induced myocarditis in BALB/c mice, the treatment with IL-15 had a positive effect on the clinical course of myocarditis, significantly improved systolic and diastolic functions of the left ventricle, and also led to a decrease in cellular infiltrates in the myocardium [120]. In addition, IL-15 has also been shown to be cardioprotective in an in vitro cell model under hypoxic conditions [121].

#### 3.2.11. Interleukin 17

It has been observed that several pro-atherogenic factors, including cholesterol, modified LDL and fatty acids, can affect the expression of IL-17 both directly and indirectly through cytokines that stimulate the secretion of IL-17. This is especially important given that IL-17 is associated with a number of autoimmune diseases, and the analysis of the mechanisms of mutual regulation of pro-atherogenic factors and IL-17 may provide insight into the pathophysiological relationship between atherosclerosis and autoimmune diseases [122]. At the same time, the exact role of IL-17 in the development of the disease and the stability of plaques remain debatable [123]. Recent studies have shown that IL-17 expression is significantly increased in patients with rheumatoid arthritis and atherosclerosis. Accumulating evidence indicates that specific pathways for cellular lipid metabolism play an important role in regulating the differentiation and function of Th17 cells, which in turn are characterized by the expression of interleukin-17 (IL-17A), IL17F, interleukin-6 (IL-6), TNF-α and interleukin-22 (IL-22) [124].

#### 3.2.12. Interleukin 22

IL-22 is a member of the IL-10 family, is associated with Th17, and is involved in autoimmune diseases including lupus and rheumatoid arthritis [125]. IL-22 expression has been found in human atherosclerotic plaques in the carotid arteries, and elevated levels have been found especially in patients with unstable plaques [126]. IL-22 expression has also been confirmed in a variety of inflammatory cell types, including macrophages and T cells, as well as in vascular smooth muscle cells (VSMC), further indicating a role in atherosclerosis [127]. Recently, numerous studies showed that IL-22 is involved in the pathogenesis of atherosclerosis by regulating VSMC proliferation and migration, angiogenesis, inflammatory response, hypertension, and cholesterol metabolism. The exact role of IL-22 in atherosclerosis is still controversial, although most studies point to the pro-atherogenic function of IL-22. Mice with apoE/IL-22 double knockout detects reduced plaque size in both the aortic root and the aorta compared to the control group knocked out by apoE. Moreover, in another study of atherosclerosis, IL-22R1 and IL-22 are expressed in atherosclerotic plaques of mice, and their expression levels are significantly increased in mice with apoE knockout [128].

### 3.3. Impact of the Tumor Necrosis Factor Superfamily (TNFSF) on Cholesterol Metabolism

#### 3.3.1. TNF-α

The main representative of the TNFSF family can be considered TNF-α. This main cytokine of the immune response is pro-atherosclerotic due to its pro-inflammatory action. According to the classical representation of Th1 cytokines such as TNF-α, IFN-γ and IL-12 play a pro-atherogenic role. A large number of studies indicate a decrease in the rate of development of atherosclerosis with a deficiency of TNF-α. It has also been shown that the use of TNF-α blockers in complex therapy causes a decrease in the frequency of initiation of cardiovascular events in patients with rheumatoid arthritis (RA), the high density lipoproteins (HDL) cholesterol content increases and the level of CRP and IL-6 decreases after 2 weeks of use. At the same time, long-term therapy has the opposite effect on the level of lipoproteins, forming a pro-atherogenic profile [129]. Clinically significant elevations of Thl cytokines (IFN-γ, endotoxin, TNF-α, and IL-1β) above baseline disrupt normal cholesterol reverse transport (OTC) and cholesterol outflow and are associated with a higher risk of coronary heart disease (CHD), acute myocardial infarction (MI), and heart failure [130,131]. In addition, it was noted that excessive levels of TNF-α in coronary heart disease are associated with increased uptake of modified LDL by macrophages, with a characteristic increase in the expression of scavenger receptors [132].

#### 3.3.2. TRAIL

TRAIL, the next representative of TNFSF that we will mention in this review, is responsible for controlling several vital processes: vasodilation, angiogenesis and inflammation. And it is interesting to note that adjustable TRAIL effects can have both pro-atherogenic effects [133], and anti-atherogenic effect [134,135]. Overall, it should be noted that in most studies, increases in MCP-1, IL-1β, and VCAM-1 levels were observed with TRAIL deficiency rather than with increased expression [136,137]. At the same time, there are works challenging this statement [138]. A series of clinical studies confirms the protective role of TRAIL. TRAIL levels have been shown to be reduced in patients with CHD. And at the same time, In CHIANTI showed that low levels of TRAIL concentrations were associated with nearly twice the risk of cardiovascular death compared to patients with high TRAIL levels [139,140]. This suggests that TRAIL levels may act as a good predictor of the risk of death in patients with stable angina, stroke and some other CVD.

### 3.4. Impact of Chemokines on Cholesterol Metabolism

The role of chemokines is intriguingly diverse. For example, a large body of evidence suggests that CC-chemokines regulate angiogenesis caused by inflammation, while angiogenesis caused by ischemia does so without the participation of CC-chemokines. Differential regulation of angiogenesis with CC-chemokines may provide an alternative strategy for the treatment of pathological diseases associated with angiogenesis [141]. C-C motif ligand 2/Monocyte Chemoattractant Protein 1 (CCL2/MCP-1) coordinates the movement of inflammatory monocytes between bone marrow, blood, and atherosclerotic plaques by binding to its related CCR2 receptor [142]. In the studies, increased expression of another representative of this group of regulatory molecules was found: chemokine (C-C motif) ligand 1 (CCL1) in the aorta of mice prone to atherosclerosis, in addition, CCL1 provided recruitment of leukocytes to the lesion [143]. In mice dually deficient in CCL1 and ApoE, an increase in atherogenesis was observed, which was also associated with a decrease in plasma IL-10 levels, a shift in the Th1/Th2 ratio towards Th1 in the spleen, and a decrease in the number of regulatory T cells in the aorta and spleen. In addition, a decrease in regulatory T cells was observed in the aorta of mice treated with CCR8-blocking antibodies and was associated with exacerbation of atherosclerosis. Thus, disturbances in CCL1-CCR8 can inhibit the production of IL-10, reduce the number and functions of regulatory T cells, which leads to the development of atherosclerosis [144].

### 3.5. Impact of Colony-Stimulating Factors (CSF) on Cholesterol Metabolism

Colony-stimulating factors (CSFs) are glycosylated cytokines that are produced and secreted by a number of different cell types, including immune and non-immune cells. Initially, these factors were identified based on their in vitro ability to facilitate the differentiation and survival of hematopoietic precursors into individual immune cell lines, as indicated by their names. For example, granulocytes colony-stimulating factor (G-CSF) promotes neutrophil differentiation, proliferation, and survival; M-CSF promotes the development of monocytes and macrophages from hematopoietic precursors; granulocyte-macrophage colony-stimulating factor (GM-CSF) affects the differentiation of stem cells into monocyte and granulocyte lines, including neutrophils, eosinophils and basophils; interleukin-3 (IL-3) or multi-colony-stimulating factor (multi-CSF) promotes the differentiation of multipotent hematopoietic stem cells into myeloid and lymphoid lines. However, it is becoming increasingly clear that these CSFs play a role in the specification and development of immune cell clones beyond their intended function [145].

In atherosclerotic lesions, blood-derived monocytes differentiate into distinct macrophage subpopulations, and further into cholesterol-filled foam cells under a complex milieu of cytokines, which also contains M-CSF and GM-CSF. One study generated human macrophages in the presence of either M-CSF or GM-CSF to obtain M-MØ and GM-MØ, respectively. The macrophages were converted into cholesterol-loaded foam cells by incubating them with acetyl-LDL, and their atheroinflammatory gene expression profiles were then assessed. Compared to GM-MØ, the M-MØ expressed higher levels of CD36, SRA1, and ACAT1, and also exhibited a greater ability to take up acetyl-LDL, esterify cholesterol, and become converted to foam cells. M-MØ foam cells expressed higher levels of ABCA1 and ABCG1 (ATP-binding cassette transporter G1), and, correspondingly, exhibited higher rates of cholesterol efflux to apoA-I and HDL2 [146].

### 3.6. Impact of Transforming Growth Factors (TGF) on Cholesterol Metabolism

Β transforming growth factor (TGF-β) is a strongly pleiotropic cytokine that exists in three isoforms in mammals (TGF-β1, TGF-β2, and TGF-β3). The importance of TGF-β is due to the fact that it contributes to apoptosis control, angiogenesis, wound healing, immune regulation, and tumor biology. Virtually all cells have receptors for TGF β, and at least one of the isoforms is produced in all tissues. Immune system cells produce predominantly TGF-β1. TGF-β is also commonly found in plasma (an isoform of TGF-β1) and is bound to extracellular matrix proteins throughout the body. It is noteworthy that platelets and bones contain large amounts of TGF-β1 [147]. Endothelial TGF-β signaling is one of the main factors causing vascular inflammation associated with atherosclerosis. The ongoing inflammation leads to activation of TGF-β signaling and induces the so-called endothelial-mesenchymal transition (EndMT), which leads to pro-atherogenic consequences: increased expression of adhesion molecules ICAM-1 and VCAM-1, further influx of inflammatory cells, fibronectin deposition, the appearance of new mesenchymal cells that give rise to SMCs and fibroblasts [148]. The inhibition of endothelial TGF-β signaling in mice with hyperlipidemia reduces inflammation in the vascular wall and vascular permeability and leads to a halt in the progression of the disease and regression of existing lesions. These effects of endothelial TGFβ signaling stand in stark contrast to its effects in other cell types and identify it as an important growth factor in atherosclerotic plaques and demonstrate some potential for therapeutic intervention [149]. For example, the mechanism of the protective effect of ApoA-I on endothelial function through the inhibition of EndMT induced by TGF-β1 has been shown, which can be further used as a therapeutic target in the treatment of atherosclerosis [150].

Table 1 shows the main effects of the cytokines described above.

## 4. Conclusions

Atherosclerosis is now recognized as an inflammatory condition thanks to decades of research. New approaches to the treatment of this pathology are the use of anti-inflammatory strategies. It is worth noting that modern anti-inflammatory drugs should target atherosclerosis-specific immune mechanisms with a minimum of systemic side effects. Therapy targeting the inflammatory-interleukin-1-interleukin-6 pathway has been successful. However, patients experience systemic health problems due to the complexity of the effects on regulating the secretion of these interleukins.

Our research group have a special interest in the field of the investigation of interleukin impacts on the atherosclerosis progression and we aim at summarizing all the known facts about this. Summing up this review, it should be noted that a large number of cytokines involved in inflammatory signaling pathways are involved in the processes of atherogenesis. These cytokines are produced by cells of the immune system and activate signaling pathways and genes involved in the metabolism of cholesterol, which is the main participant in the formation of atherosclerotic plaques. To date, not all mechanisms of cytokine involvement in the pathogenesis of atherosclerosis have been fully studied, which represents a large field for further research. However, it can be concluded that some cytokines can be considered as promising targets for the treatment and prevention of atherosclerosis.

## Figures and Tables

**Table 1:** The main effects of the described cytokines.

Group	Action Molecules	Secreting Cells	Effector Cells	Effects
TNF Super Family	TNF-a	Macrophages, lymphoid cells, mast cells, endothelial cells, cardiac myocytes, adipose tissue, fibroblasts and neurons.	Macrophages, monocytes, B cells, T cells, NK cells, endothelial cells.	Stimulates phagocytosis, production of IL-1 oxidants and the inflammatory lipid prostaglandin E2 (PGE2) [151], IL-10 production, proliferation, Ig production, HLA-DR and CD25 expression, GM-CSF production [152], enhancement of cytotoxic activity, cell death, induction of pro-coagulant agents [153], adhesion molecules and pro-inflammatory cytokines [154].
Interferons	IFN-I	Fibroblasts and monocytes.	Natural killer cells and macrophages.	Activate immune cells, increase host defenses by up-regulating antigen presentation by virtue of increasing the expression of major histocompatibility complex (MHC) antigens [155].
	IFN-II	Adaptive immune cells, more specifically CD4+ T helper 1 (Th1) cells, natural killer (NK) cells, and CD8+ cytotoxic T cells.	Macrophages, B cells, CD8+ cytotoxic T cells.	Promote inflammation, antiviral or antibacterial activity, and cell proliferation and differentiation [156].
	IFN-III	Type 2 myeloid dendritic cells.	Epithelial cells, neutrophils, B cells and dendritic cells.	Modulate the immune response after a pathogen has been sensed in the organism, their functions are mostly anti-viral and anti-proliferative [157].
Chemokines	CC	Cells of innate and adaptive immunity.	T cells, eosinophils and basophils, monocytes, NK cells and dendritic cells.	Induces monocytes to leave the bloodstream and enter the surrounding tissue to become tissue macrophages, induce the migration of monocytes and other cell types such as NK cells and dendritic cells [158].
	CXC		Neutrophils, lymphocytes.	Induces the migration of neutrophils, activating their metabolic and degranulation [159], chemoattractant for lymphocytes [160].
	C		T cell, dendritic cells.	Involved in cross-presentation, antigen uptake, and induction of innate as well as adaptive cytotoxic immunity, to increate T cells in joints that are affected with rheumatoid arthritis [76].
	CX3C		T cell, monocytes, leukocytes.	Soluble, potently chemoattracts T cells and monocytes [77], cell-bound chemokine promotes strong adhesion of leukocytes to activated endothelial cells.
CSF	CSF1	Different types of cells.	Hematopoietic stem cells, monocytes, macrophages.	Causes hematopoietic stem cells to differentiate into macrophages or other related cell types [161].
	CSF2	Macrophages, T cells, mast cells, natural killer cells, endothelial cells and fibroblasts.	Affects more cell types, especially macrophages and eosinophils.	Stimulates stem cells to produce granulocytes (neutrophils, eosinophils, basophils) and monocytes. Activates the maturation of monocytes and dendritic cells [152].
	CSF3	Endothelium, macrophages, and a number of other immune cells.	Precursor cells in the bone marrow, neutrophil precursors and mature neutrophils, hematopoietic stem cell.	Stimulates the bone marrow to produce granulocytes and stem cells and release them into the bloodstream, stimulates the survival, proliferation, differentiation, and function of neutrophil precursors and mature neutrophils.
TGF	TGFα	Macrophages, brain cells and keratinocytes.	Epithelial cells, neural cell.	Induces epithelial development, stimulates neural cell proliferation [162].
	TGFβ	All white blood cell lineages.	Macrophages, stem cell, T cell, B cell.	Plays crucial roles in tissue regeneration, cell differentiation [149], embryonic development and regulation of the immune system [163].
IL-1 family	IL-1β	Activated macrophages.	Different cell types.	Important mediator of the inflammatory response [164], and is involved in a variety of cellular activities [94], including cell proliferation, differentiation and apoptosis [53].
	IL-4	Mast cells, Th2 cells, eosinophils and basophils.	B cell and T cell, macrophages.	Induces differentiation of naive helper T cells (Th0 cells) to Th2 cells [165], promotes alternative activation of macrophages into M2 cells and inhibits classical activation of macrophages into M1 cells [166].
	IL-5	Type-2 T helper cells and mast cells.	B cell, eosinophils.	Stimulates B cell growth and increases immunoglobulin secretion, mediator in eosinophil activation.
	IL-6	Macrophages, osteoblasts, smooth muscle cells.	Neutrophils, B cells, T cells.	Stimulating acute phase protein synthesis [167], as well as the production of neutrophils in the bone marrow [168], it supports the growth of B cells and is antagonistic to regulatory T cells.
	IL-7	Stromal cells in the bone marrow and thymus, keratinocytes, dendritic cells, hepatocytes, neurons and epithelial cells.	B cells, T cells and NK cells.	Stimulates the differentiation of multipotent (pluripotent) hematopoietic stem cells into lymphoid progenitor cells [169], stimulates proliferation of all cells in the lymphoid lineage [170].
IL-8 is a member of the CXC family of chemokines	IL-8	Macrophages and other cell types.	Granulocytes.	Induces chemotaxis in target cells, primarily neutrophils, stimulates phagocytosis, promoter of angiogenesis [171].
	IL-10	Monocytes and, to a lesser extent, lymphocytes.	Th1, Macrophages, B cell.	It downregulates the expression of Th1 cytokines, MHC class II antigens, and co-stimulatory molecules on macrophages. It also enhances B cell survival, proliferation, and antibody production. IL-10 can block NF-κB activity, and is involved in the regulation of the JAK-STAT signaling pathway [172].
IL-12 family	IL-12	Dendritic cells, macrophages, neutrophils, and human B- lymphoblastoid cells (NC-37).	T cells, NK cells.	Stimulates the growth and function of T cells [173], stimulates the production of interferon-gamma (IFN-γ) and tumor necrosis factor-alpha (TNF-α) from T cells and NK cells, and reduces IL-4 mediated suppression of IFN-γ, block the formation of new blood vessels [117].
	IL-13	Th2 cells, CD4 cells, natural killer T cell, mast cells, basophils, eosinophils and nuocytes.	Hematopoietic cells, B cell.	Regulator in IgE synthesis, goblet cell hyperplasia, mucus hypersecretion, airway hyperresponsiveness, fibrosis and chitinase up-regulation [174].
	IL-15	Mononuclear phagocytes.	NK cells, T cells.	Induces the proliferation of natural killer cells [175].
IL17 family	IL-17A	Activated T cells.	Th17.	Regulates the activities of NF-kappaB and mitogen-activated protein kinases, stimulate the expression of IL6 and cyclooxygenase-2 (PTGS2/COX-2), as well as enhances the production of nitric oxide (NO).
	IL-22	Tissue cells, αβ T cells classes Th1, Th22 and Th17 along with γδ T cells, NKT, ILC3, neutrophils and macrophages.	Non-hematopoietic cells—mainly stromal and epithelial cells.	Stimulation of cell survival, proliferation and synthesis of antimicrobials [176].

## Data Availability

Not applicable.
